# Identification of protein-protein and ribonucleoprotein complexes containing Hfq

**DOI:** 10.1038/s41598-019-50562-w

**Published:** 2019-10-01

**Authors:** Joël Caillet, Bruno Baron, Irina V. Boni, Célia Caillet-Saguy, Eliane Hajnsdorf

**Affiliations:** 1 UMR 8261, CNRS, Université de Paris, Institut de Biologie Physico-Chimique, 75005 Paris, France; 2 Institut Pasteur, Plateforme de Biophysique Macromoléculaire, Center for Technical Resources and Research (C2RT), 25–28 rue du Dr Roux, 75724 Paris, cedex 15 France; 3 Shemyakin-Ovchinnikov Institute of Bioorganic Chemistry RAS, 117997 Moscow, Russia; 4 Institut Pasteur, Laboratoire Récepteurs-Canaux, Département de Neuroscience, 25–28 rue du Dr Roux, 75724 Paris, cedex 15 France

**Keywords:** RNA-binding proteins, Isolation, separation and purification

## Abstract

Hfq is a RNA-binding protein that plays a pivotal role in the control of gene expression in bacteria by stabilizing sRNAs and facilitating their pairing with multiple target mRNAs. It has already been shown that Hfq, directly or indirectly, interacts with many proteins: RNase E, Rho, poly(A)polymerase, RNA polymerase… In order to detect more Hfq-related protein-protein interactions we have used two approaches, TAP-tag combined with RNase A treatment to access the role of RNA in these complexes, and protein-protein crosslinking, which freezes protein-protein complexes formed *in vivo*. In addition, we have performed microscale thermophoresis to evaluate the role of RNA in some of the complexes detected and used far-western blotting to confirm some protein-protein interactions. Taken together, the results show unambiguously a direct interaction between Hfq and EF-Tu. However a very large number of the interactions of proteins with Hfq in *E. coli* involve RNAs. These RNAs together with the interacting protein, may play an active role in the formation of Hfq-containing complexes with previously unforeseen implications for the riboregulatory functions of Hfq.

## Introduction

In all organisms, RNA-binding proteins participate in modulating all the steps in the life cycle of RNA, including transcription, folding, translation and turnover. Hfq is a RNA-binding protein playing a pivotal role in the control of gene expression in bacteria. In *E. coli*, this RNA chaperon is involved in many cellular processes controlled by small non-coding RNAs (sRNAs), including modulation of RNA transcription, translation and degradation^1,2^. A gene for the Hfq protein is present in half of the sequenced bacterial genomes including many pathogens^3,4^, though no Hfq orthologs have been found in Actinobacteria. Hfq was shown to act as a virulence factor in several bacterial pathogens^5^, but it is not clear whether it can be considered as a general factor required for the virulence of all Hfq-containing pathogens. Hfq also has DNA-binding activity: it is classed as one of the twelve nucleoid-associated *E. coli* proteins, and, in addition, it induces negative supercoiling into plasmid DNA^6–8^.

Hfq binding stabilizes sRNAs and facilitates their pairing with multiple target mRNAs, thereby controlling their translation either positively or negatively^9^. Base pairing with sRNAs also frequently affects the half-life of mRNA targets, which can either be stabilized or become more prone to degradation. Hfq is one of the proteins interacting with RNase E^10^, and the recruitment of RNase E by Hfq leads to its targeting of some mRNA-sRNA hybrids and to their rapid degradation by RNase E^11–13^. Besides RNase E, Hfq has also been reported to associate with Rho, poly(A)polymerase (PAP), and RNA polymerase^14–16^.

Many sensitive high-throughput techniques have been developed to define protein-protein interactions. Systematic analysis of protein-protein interactions by pull-down assays and/or co-purification in *E. coli* has revealed that Hfq interacts with numerous proteins including interactions with subunits of RNA polymerase, RNase E and other degradosome components^17,18^. The web site http://www.ebi.ac.uk/intact/main.xhtml provides an open source database and analysis tools for protein interactions mainly based on the systematic analyses of protein-protein interactions. First, the ASKA His-tagged ORF clone library was used in large-scale pull-down assays, and proteins co-purifying with His-tagged baits on Ni^2+^-NTA column were identified by MALDI-TOF MS^18^. In the second method, Sequential Peptide Affinity (SPA) or Tandem-Affinity-Purification (TAP)-tagged derivatives were used to isolate the interacting protein partners using two rounds of affinity chromatography, which were then identified by MS^17^. Overall 79 interactants were found to bind Hfq, but somewhat worryingly, only two interactants are common to both sets of data; the ribosomal proteins RpsD and RplB. Moreover, further analysis of these data showed that most interactions originated from spoke-expanded co-complexes (*i.e* the prey protein is part of a complex which interacts with the Hfq bait protein). After filtering so that the prey protein interactions with all other proteins of the complex were removed from the matrix model, binary complexes were reduced to 2, Hfq with itself and Hfq with Rho. In addition, a recent resource details a global landscape of cell envelope protein complexes in *E. coli*. Hfq appears in 3 complexes: two large complexes comprising 83 and 48 subunits and a smaller complex of 7 subunits (BamD, RluC, LptA, MetC, RppH, TamA and Hfq)^19^. Overall these results strongly imply that Hfq is a “sticky” protein capable of multiple interactions, which may not all be specific.

Characterizing protein-protein interactions is challenging in the case of Hfq as the previously used global approaches can be prone to artifacts. In particular Hfq is one of the proteins exhibiting a false-positive binding activity during Ni-affinity purification^20^. In addition, due to its very high affinity for RNA, Hfq purification requires very stringent conditions to ensure RNA elimination^7,21–23^.

The main goal of this study was to exploit alternative approaches to confirm previously identified Hfq-interacting proteins, to search for new interacting proteins and to determine whether their interactions with Hfq are mediated by RNA. To this purpose, we have used TAP-tag purification combined with RNase A treatment, and protein-protein crosslinking, which freezes protein-protein complexes formed *in vivo*. Altogether 246 non-ribosomal proteins were found to interact with Hfq with 55 detected by the two approaches. Since RNase A treatment disrupts the interaction of 42 out of the 141 complexes identified after TAP-tag purification, our results suggest that most of the interactions of proteins with Hfq in *E. coli* are mediated through RNA or involve RNA. Interestingly we show that RNA may stabilize the Hfq-protein interaction or compete with the other partner to interact with Hfq as shown for Rho or poly(A)polymerase.

## Material and Methods

### Strains and plasmids

The strains and plasmids used in this study and their constructions are described in Table S1.

### Preparation of the extracts for TAP-tag experiments

Strain IBhfq95Δ*hfq* was transformed by the plasmids pHfqTT, a derivative of pTX381, expressing Hfq with the TAP-tag (HfqTT) from the *hfq* promoter and by pCATT as a control. In this latter case the TAP-tag peptide is expressed from an IPTG inducible promoter by addition of 5 10^−5^ M IPTG (Table S1). This concentration was chosen because it allows HfqTT transcribed from the inducible T5-lac promoter (IBhfq95 Δ*hfq*pCAhfqTT) to control *ptsG* expression as efficiently as when it is expressed from its own promoter (IBhfq95Δ*hfq* phfqTT) (Table S2B). Bacteria were grown in LB at 37 °C and harvested at A_650_ of 0.6 (exponential phase) and after 16 h (stationary phase). Extracts were prepared essentially as described in^24^ with the following modifications. Cells, resuspended in lysis buffer, were passed through a French press (1200 bar, 20000 psi). The cell extracts were clarified by centrifugation and the supernatants submitted to two rounds of affinity chromatography. Half of each sample was treated with 0.1 µg/µl RNase A and incubated for 30 min at 37 °C, after the IgG Sepharose beads column and TEV cleavage and before the affinity purification on calmodulin beads. The extracts were then analysed by mass spectrometry by the “Plate-forme Protéomique Paris 5 (3P5) Descartes”.

### *In vivo* DSP crosslinking and enrichment in Hfq-interacting proteins

Strain IBhfq95Δ*hfq* was transformed by the plasmids pHfqH6, a pACYC184 derivative expressing HfqH6, or pACYC184, as a control (Table S1). Cultures in LB medium were harvested in late exponential phase (A_650_ = 1), pellets were washed twice in PBS and resuspended in 0.25 mM DSP (Dithiobis[succinimidyl propionate) in PBS^25^ for 1 h at 37 °C. The reaction was then stopped by addition of 50 mM Tris pH 8. The suspensions were passed through a French press. The cell extracts were clarified by centrifugation and the supernatants submitted to Ni-Agarose affinity chromatography as indicated by the supplier (wash in 20 mM Tris pH 7.5, 300 mM NaCl, 20 mM imidazole and elution in 20 mM Tris pH 7.5, 300 mM NaCl, 300 mM imidazole). Half of each extract was incubated with 50 mM DTT at 37 °C for 1 h (without any RNase A treatment) to reverse the crosslinking before loading on 10% SDS-PAGE gel. Gels were stained with colloidal blue. Each lane was cut into 24 slices, which were directly analysed by mass spectrometry (see supplementary Material and Methods) by the proteomic platform of Strasbourg Esplanade.

### Purification of proteins, far-western, DSC, CD and MST experiments

Ec-HfqHis6 was overexpressed in a ∆*hfq* strain, purified as described previously^23^ and stored in standard buffer (50 mM Tris-HCl pH7.6, 50 mM NH_4_Cl, 1 mM EDTA, 0.1% Triton X100, 5% glycerol). We verified the thermal stability of Hfq by Differential Scanning fluorimetry (NanoDSF) using Prometheus (Nanotemper GmbH), and by Differential Scanning Calorimetry (DSC) using CapDSC (Malvern). Both experiments were performed in standard buffer from 25 to 95 °C for NanoDSF and from 30 to 110 °C for DSC, and at a rate of 1 °C/min. In both cases, no denaturation was observed. Circular dichroism (CD) experiments in the far UV confirmed moreover that the protein was correctly folded, exhibiting a typical mixed alpha/beta structure (data not shown). Rho was a gift of Marc Boudvillain (CNRS UPR4301). RNA polymerase was overproduced from the multicopy plasmid pVS10 (T7P-α–β–β′-His6–ω) with a C-terminally His-tagged ß′ subunit (gift of Nicolas Joly, Institut Jacques Monod) in a ∆*hfq* strain and purified as in^26^. His-tagged EF-Tu was a gift of Sophie Chat (CNRS UMR6290). It was overexpressed in *E. coli* BL21(DE3) and then isolated using Ni^2+^ pre-charged HiTrap chelating columns. Ribosomal protein S1 was purified as in^22^ after washing the poly(A) Sepharose column with buffer containing 1 M NH_4_Cl. The eluted material was passed through a G100 Sephadex column in the same buffer, and the fractions containing S1 were concentrated. PAP was purified as in^27^. Total RNA was extracted as in^28^. Poly(A) and poly(G) were from Sigma.

Far western blot was performed as described in^29^. Briefly, purified proteins were run on 12.5% SDS-PAGE (Acrylamide/bisacrylamide 29:1) and transferred to nitrocellulose blotting membrane (AmershamTM ProtanTM 0.45 µm NC). The proteins on the membrane were denaturated in buffers with varying concentrations of guanidine-HCl and then renaturated by overnight incubation at 4 °C in 20 ml protein-binding buffer containing (far western) or not (western) purified Hfq (20 µg). After washing, bound Hfq was detected with an anti-Hfq polyclonal antibody^23^ and an anti-rabbit IgG(H + L) HRP-conjugated secondary antibody using BioRad ClarityTM Western ECL Substrate and digital imaging.

For MST experiments, the proteins of interest were labeled with a fluorescent dye reactive on amine or cysteine (Protein Labeling Kit RED-NHS, Nanotemper GmbH). A serial dilution of titrant was prepared in standard buffer. The concentration of the labeled molecule was constant and the concentration of the interactant varied. An equal volume of the serial dilution and of the diluted labeled molecule were mixed and loaded in glass capillaries. The MST measurements of the binding affinity of Hfq for the interactants were performed on a Monolith NT.115 (NanoTemper, Gmbh), with 80% LED and 20% MST power at room temperature. The specific change in the thermophoretic mobility upon titration is measured as a delta of normalized fluorescence. The data analysis was performed with NT Affinity Analysis software.

## Results

Our goal was to clarify the situation concerning RNA in Hfq interactions and to identify other direct protein-protein interactions with Hfq as a partner. Two approaches were used: (i) the Tandem-Affinity-Purification (TAP-tag) which allows the rapid purification of complexes under gentle native conditions, including (or not) an RNase A treatment to improve the specificity and to distinguish protein- and RNA-mediated interactions and (ii) an *in vivo* chemical crosslinking method by using Dithiobis[succinimidyl propionate] (DSP). To this end, strain IBhfq95Δ*hfq* mutant, was transformed with low copy number plasmids^23^ expressing the genes encoding HfqTT (TAP-tagged) or HfqH6 (His6) proteins (both tags at the C-terminus). These proteins were expressed from the native *hfq* promoter (Table S1) to conserve the biologically relevant yield of Hfq by maintaining the auto-control of *hfq* expression^23,30^. The Hfq proteins, with or without the TAP-tag, expressed from the plasmids were able to control the expression of the *rpoS::lacZ* fusion and to complement the Δ*hfq* mutation, which was shown by their ability to restore growth of the *hfq* mutant strain on plates containing α-methyl glucoside similarly to wt Hfq^31^ (Table S2). The carboxy terminal domain of Hfq was shown to limit sRNA-mRNA association^32^. Our data suggest that the tag at the C-terminus of the protein does not strongly perturb its functions *in vivo*.

### The TAP-tag experiments

The Δ*hfq* mutant strain was transformed with the pHfqTT plasmid, encoding the TAP-tagged Hfq, or with the control pCATT plasmid, encoding the TAP-tag peptide induced by IPTG. Cultures were harvested in exponential and stationary phase since Hfq is maintained at a level characteristic of the rate of cell growth^33^. Proteins, which were detected in the TAP-tag-only control experiment with the pCATT plasmid including H-NS, SecA, enolase, NifU and numerous ribosomal proteins, were not further taken into account (Table S3, sheet 2). We should emphasize here that in the previous work exploiting the TAP-tag technique^17^, the interacting protein partners were isolated using two rounds of affinity chromatography without any RNase treatment. In the present work, to distinguish which interactions require RNA, the protein complexes were isolated first by affinity chromatography on IgG Sepharose beads followed by TEV cleavage and then half of each sample was treated with RNase A at a high concentration, before passing through a calmodulin affinity column. Table S3 Sheet 1 lists all the proteins confidently identified by Mass Spectrometry. We considered a protein as a valid candidate partner if it was identified by at least two peptides in any one of the conditions (exponential or stationary +/− RNase A) as identified by LTQ-Orbitrap MS/MS with a Mascot score over threshold (coloured in olive green in Table S3 sheet 1). As expected Hfq scores highly in both the + and − RNase A conditions, although there is about a 30% decrease in score + RNase A in both exponential and stationary. Since Hfq harbors the TAP-tag, the number of peptides identified either in exponential phase or in stationary phase was not expected to vary as a consequence of RNase A treatment. We have used the number of Hfq self-interactions, plus or minus RNase A treatment, as an internal control to distinguish the RNA dependence of the interaction and to compare the number of peptides identified in samples treated or untreated with RNase A in each growth condition. Based on this analysis, an interaction was considered as dependent on RNA when the number of peptides decreased after the RNase A treatment by more than 30%. The high RNase A concentration used in this treatment allows us to consider that all the accessible RNA was degraded. It can be noted that for many of the proteins that we classify as RNA independent, the score of peptides after RNase A treatment actually increases markedly (Table S3, sheets 1 and 4).

Several classes of Hfq-interacting proteins may be distinguished, depending on their sensitivity to RNase treatment and on the growth phase. However it can be noticed that an overwhelming majority of the candidate Hfq partners are present in a similar amount in both exponential and stationary phases (Table S3 sheet 1).

The TAP-tag purification identified 141 single species candidates as potential Hfq interactants including 45 ribosomal proteins, which were not further taken into account. Of the 96 remaining interactants, 42 are considered as RNA-dependent and 59 as not RNA-dependent according to our criteria (Table S3, sheets 3 and 4). Three candidates (Lon, RpoB and TnaA) are classified as RNA dependent in exponential phase but RNA independent in stationary phase.

### *In vivo* crosslinking and enrichment for Hfq interactants

**Figure 1:**
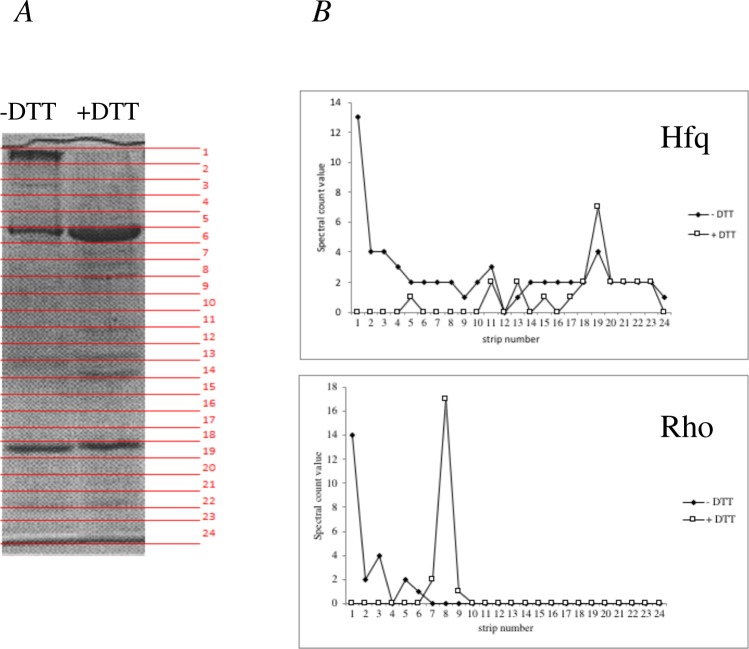
*In vivo* DSP crosslinking and analysis of Hfq-containing complexes. IBhfq95Δ*hfq* cells containing the pHfqH6 plasmid were treated with DSP, followed by lysis and enrichment of HfqH6 containing complexes on Ni-NTA Agarose columns. The extracts column fractions were submitted to DTT treatment, or not, before separating on SDS PAGE. The figure shows the same fraction without and with DTT treatment. The full-width gel with negative controls is presented in Supplementary Fig. 1. Note that DTT treatment eliminates the high molecular weight aggregates with an increase in Hfq hexamer (strip #6) and range of other proteins of different molecular weights. (**A**) Each lane was cut into 24 strips from the top to the bottom of the gel, which were directly subjected to MS analysis. (**B**) Profiles of Hfq and Rho after DTT treatment (empty/open symbols) or not (black/filled symbols) are shown as a Spectral count value taken as a measure of the abundance of each protein in function of the strip number.

In order to capture protein-protein complexes in their native environment, the membrane permeable cross-linker dithiobis succinimidyl propionate (DSP) was added to growing IBhfq95Δ*hfq* cells containing pHfqH6 plasmid or the empty vector pACYC184^25,34^ in late exponential phase. After enrichment on Ni-NTA Agarose columns, the Hfq-crosslinked complexes were treated (or not) with DTT, to reverse the crosslinking, and loaded on SDS-PAGE gel (Fig. 1A). Each lane was cut into strips and submitted to MS analysis. We looked for candidates exhibiting a modified migration profile on the gel after treatment with DTT (Table S4 Sheet 1). As an example, the Rho protein is crosslinked to HfqH6 by DSP, so that it migrates near the top of the gel and upon addition of DTT both Hfq and Rho migrate according to their molecular weights (Fig. 1B).

**Figure 2:**
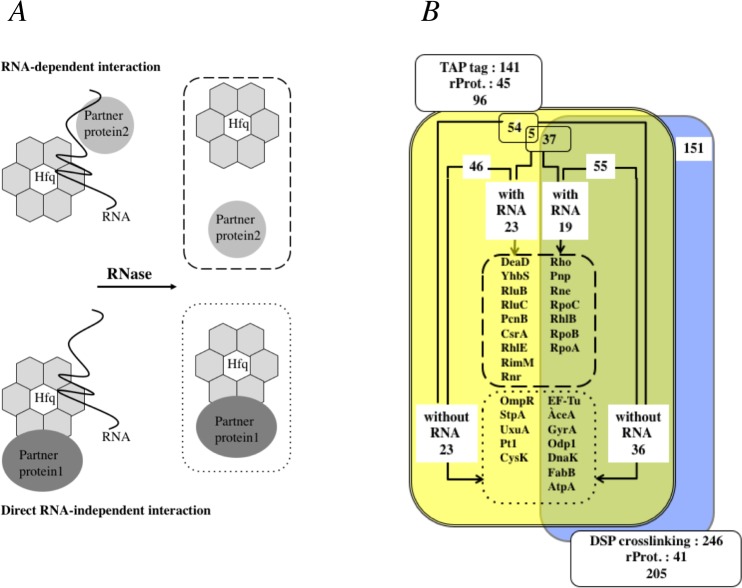
Summary of candidate Hfq-interacting proteins obtained by crosslinking and TAP-tag experiments. (**A**) Schematic representation of both RNA-dependent and RNA independent Hfq-interacting protein complexes. (**B**) The numbers of candidates obtained by the two techniques are shown within boxes: TAP-tag (double line in yellow) and cross-link (single grey line in blue) and those observed by the two techniques (total 55) in the overlapping space (in green). The highest scoring candidates (scores of 4 and over) for (i) RNA-dependent interaction with Hfq are given listed within the box with dashed lines, while the highest scoring (ii) RNA-independent interactants are given within the dotted box.

The analysis of the control sample (the empty vector pACYC184) revealed a few false positive candidates, able to bind to Ni-NTA Agarose columns in the absence of a H6 tag, such as GlmS^20^ (data not shown), which were not further taken into account. DSP crosslinking revealed 246 Hfq interactants of which 205 were non-ribosomal proteins (Table S4). Of the 96 non-ribosomal candidates found in the TAP-tag experiment in the absence of RNase A, 55 were also found in the crosslink experiments (Fig. 2). These 55 proteins thus constitute a reliable data set of Hfq-interacting proteins, and include proteins, which interact via an RNA intermediate or independently of RNA.

### Analysis of Hfq interacting protein candidates

#### RNA dependence of known Hfq interactants

The highest scores of the RNA-dependent partners of Hfq detected by TAP-tag concern essentially proteins belonging to RNA metabolism (dashed box Fig. 2B, Table 1 and Table S3 sheet 3), some of which were also found by the DSP crosslinking technique (Fig. 2, Tables S4, S5). They are ribonucleases (PNPase (Pnp), RNase E (Rne), RNase R (Rnr)), helicases (DeaD, RhlB), Poly(A)polymerase (PAP or PcnB), RNA polymerase subunits α, β, β′ (RpoA, B, C), RNA modification enzymes (RluB, RluC) and also RNA binding proteins (ProQ, CsrA and Rho). In most cases, RNase treatment reduced or even eliminated the interaction with Hfq as in the case of RNase E and PNPase (Table S3). The RNA dependence of these Hfq partners suggests either that the interaction with Hfq is mediated by RNA molecules as already shown *in vitro*^35^ and/or that the complex is stabilised by the RNA.

**Table 1:** List of non-ribosomal proteins, which interact with Hfq in the presence or in the absence of RNA as revealed by TAP-tag experiments.

Exponential phase RNA dependent		Stationary phase RNA dependent		
+	−	+	−	
Rho		Rho		Transcription termination factor Rho
DeaD				ATP-dependent RNA helicase deaD
Pnp		Pnp		Polynucleotide phosphorylase (PNPase)
Rne		Rne		Ribonuclease E
Lon			Lon	Lon protease
YhbS			YhbS	Uncharacterized N-acetyltransferase YhbS
RluB		RluB		Ribosomal large subunit pseudouridine synthase B
RpoC		RpoC		DNA-directed RNA polymerase subunit beta’
RluC		RluC		Ribosomal large subunit pseudouridine synthase C
PcnB		PcnB		Poly(A) polymerase (PAP)
CsrA		CsrA		Carbon storage regulato
RhlB		RhlB		ATP-dependent RNA helicase rhlB
RpoB		RpoB		DNA-directed RNA polymerase subunit beta
RimM		RimM		Ribosome maturation factor rimM
TnaA		TnaA		Tryptophanase
		Pss		CDP-diacylglycerol–serine O- phosphatidyltransferase
		Rnr		Ribonuclease R
		KatGI		Catalase-peroxidase 1
		FadI		3-ketoacyl-CoA thiolase
		Tig		Trigger factor
		YfiF		Uncharacterized tRNA/rRNA methyltransferase
		FadJ		Fatty acid oxidation complex subunit alpha
		CysY		Citrate synthase
	EF-Tu		EF-Tu	Elongation Factor Tu
			AceA	isocitrate lyase
	GyrA		GyrA	DNA gyrase subunit A
	Odp1		Odp1	Pyruvate deshydrogenase E1 component
	DnaK	DnaK		Chaperone protein dnaK
	LpxD		LpxD	UDP-3-O-[3-hydroxymyristoyl] glucosamine N-acyltransferase
	FabB		FabB	3-oxoacyl-[acyl-carrier-protein] synthase 1
			OmpR	Transcriptional regulatory protein ompR
	StpA		StpA	DNA-binding protein stpA
			AtpA	ATP synthase subunit alpha
			Pt1	Phosphoenolpyruvate-protein phosphotransferase
			UxuA	Mannonate dehydratase
			CysK	Cysteine synthase A
			EutB	Ethanolamine ammonia-lyase heavy chain
			HslU	ATP-dependent protease ATPase subunit hslU
			PutA	Bifunctional protein putA
			TyrR	Transcriptional regulatory protein tyrR
			AtpB	ATP synthase subunit beta
			SthA	Soluble pyridine nucleotide transhydrogenase
	SspB			Stringent starvation protein B

The proteins, which interact with HfqTT, but not with the TAP-tag, in the presence or in the absence of RNA in exponential and stationary phases, as revealed by TAP-tag experiments with the highest score numbers (≥4) are listed (see Table S3). A short description of each protein is given.

Hfq was reported to co-purify with RNA polymerase in an S1-dependent manner^16^. In agreement, complexes between Hfq and RNA polymerase subunits (RpoB, C and A) were found after TAP-tag and DSP procedures and the TAP-tag complexes were sensitive to RNase treatment. Similarly PNPase was fished by both techniques. This agrees with recent data showing that PNPase, Hfq and sRNA can form *in vitro* a ternary complex in which the ribonuclease plays a non-destructive structural role. Such ternary complexes were proposed to arise *in vivo* at least transiently^36^. In addition van Nues *et al*. reported that IsrA sRNA (McaS) associated with Hfq in a core complex with other proteins such as RNA polymerase, CsrA, S1 and ProQ^37^.

Rho, which was the only Hfq interactant remaining after filtering out the “spoke-expanded co-complexes” from the previous high-throughput experiments was found here to interact with Hfq by both TAP-tag and DSP crosslinking. The amount of Rho which interacted with Hfq was higher in exponential than in stationary phase, and in both phases was sensitive to the presence of RNase A (Table 1, Table S3 sheet 3). This strongly suggested that RNA is necessary for the interaction between Rho and Hfq, which is probably involved in stabilising the complex.

Various reports indicate a role of Hfq in the degradosome. Hfq was reported to copurify with RNase E along with a sRNA, (SgrS or RyhB were tested), but not with C-terminally truncated RNase E, incapable of forming a degradosome. Neither PNPase nor RhlB (both components of the degradosome) was required for the RNase E-Hfq interaction^13^. Moreover, in another report the sRNA MicC was clearly required *in vitro* to mediate the interaction between Hfq and the recognition core (part of the C-terminus) of RNase E^38^. We provide here further evidence that RNA plays a major role in the formation of RNase E-Hfq containing complexes since an interaction between Hfq and RNase E was detected *in vivo* by using the two approaches, and it was disrupted upon RNase treatment of the TAP-tag samples. The observation that RhlB and PNPase (Pnp) are also RNA-dependent interactants in the TAP-tag experiment and crosslinked to Hfq by DSP could be due to their proximity inside the degradosome. Enolase (Eno), which is also part of the degradosome, was only found in the DSP experiments (Table S4, sheet 1) thus confirming its proximity to Hfq in the degradosome. We also found that RNase R selectively interacts with Hfq in stationary phase in a complex involving RNA (Table 1, Table S3 sheet 3). This confirms similar results reported by Malecki *et al*. who used an RNase A treatment during RNase R-TAP purification of bacteria in stationary phase after they had been submitted to a cold shock^39^. Taken into account that RNase R was found to co-purify with RNase E in *Pseudomonas syringae*^40^ and that RNase R expression is induced in stationary phase^41^, we hypothesize that both RNase R and Hfq may participate in an alternative degradosome acting in stationary phase.

Known RNA binding proteins like ProQ and CsrA were also found interacting with Hfq in a RNA-dependent manner. YhbS, an uncharacterized putative N-acetyl transferase scored highly in the RNase dependent category (Table 1, Table S3 sheet 3) but unlike the enzymes of RNA metabolism RNase treatment only reduced the binding to HfqTT in exponential phase but not in stationary phase. It will be interesting to determine its true function and substrate and to elucidate whether this analysis is indicative of a role in RNA metabolism.

#### RNA independent interactants

Our criteria for RNA independent interactants in the TAP-tag experiment was a score reduced by no more than 30% after RNase treatment but for many proteins the number of peptides detected after RNase treatment increases considerably reflecting the loss of all the RNA dependent interactions (Table S3 sheet 4 and Table 1). This observation strongly supports their assignment to the class of proteins which bind Hfq independently of RNA. We note that the RNA independent interactions are more numerous in stationary phase. One possible explanation is that, in exponential phase, Hfq is interacting with sRNAs and/or with mRNA to control translation and that the increase in RNA independent interactions in stationary phase may reflect lack of competing ribosomes and a decrease in sRNA control of translation. The highest score of the direct interactants with Hfq was obtained for the elongation factor Tu (EF-Tu), one of the most abundant proteins in the cell. EF-Tu was also found as a Hfq partner by DSP crosslinking making a direct interaction with Hfq very likely. EF-Tu is a known RNA binding protein that mediates the formation of the ternary complex with aa-tRNA and GTP. Moreover, EF-Tu with Hfq, S1 and EF-Ts, are involved in RNA replication since they form part the Qβ replicase complex^42^.

Another class of interacting proteins are GyrA, HUα and β (DbhA and B), StpA, and RecA which represent DNA binding or histone-like proteins and thus confirms previous reports of Hfq binding the chromosome^6,8,43^. Both GyrA and RecA were among the interactants detected by both techniques as was the heat shock factor, protein chaperone, DnaK. The Hfq protein is thermostable, therefore it is possible that its interaction with DnaK is related to its chaperon function to assist the folding of nascent proteins.

Other proteins found to be directly interacting with Hfq included several enzymes (e.g. AceA, Odp1, FabB) (Table S5). Two subunits of the membrane associated ATP synthase, ATPA and ATPB, were found by both techniques but, almost exclusively in stationary phase. Two other subunits ATPD and ATPF were also found by crosslinking to Hfq (Table S5). Hfq has been located close to the membrane by electron microscopy^44^. It is worth noting that these RNA-independent candidates are particularly present in stationary phase which may reflect an unsuspected function of Hfq in conditions where it is released from nucleic acids, as in stationary phase.

Ribosomal proteins were strongly represented amongst the interactants with Hfq after crosslinking and in the TAP-tag experiment, but they were also found in the TAP-tag-only control, suggesting they are false-positives. Moreover, nearly all the ribosomal proteins were found associated with Hfq, not just those near the mRNA binding site. These interactions could be a non-specific consequence of the abundance of ribosomes in the cell. However it should be remembered that Hfq was initially reported as being associated with ribosomes^7^, and via its association with sRNAs, it plays a role in controlling translation initiation by binding at or near the ribosome-binding site. In addition Andrade *et al*. very recently reported that Hfq could act as a novel auxiliary ribosome biogenesis factor^45^ making a functional interaction between Hfq and ribosomal proteins possible.

The interactions of Hfq with PAP, RNA polymerase, EF-Tu, S1 and Rho were further investigated by using far western analysis and Microscale Thermophoresis (MST) in order to clarify the role of RNA in these interactions.

### Direct demonstration of binary Hfq interactions

**Figure 3:**
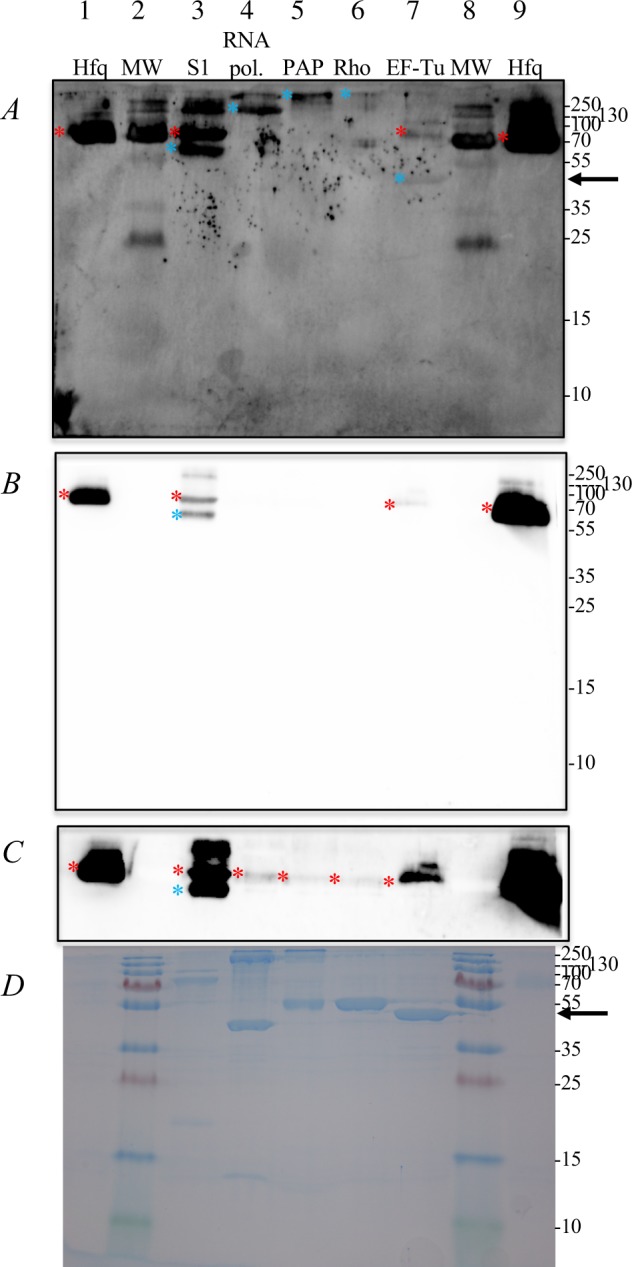
Detecting protein-protein interactions by far western blotting. The purified proteins indicated above each lane were separated on three identical SDS PAGE (12.5%) blotted to nitrocellulose membranes (**A**–**C**) or (**D**) stained by Instant Blue (Expedeon), a Coomassie based staining solution. (**A**) Far western blot, membrane was incubated with Hfq prior to treatment with Hfq antibodies. (**B**) Western blot direct detection with Hfq antibodies. Parameters for gel imaging are identical in panels A and B. (**C**) Same western blot as in (**B**) but with a longer exposure. Lanes 1, 9: 10 ng and 0.1 µg respectively Hfq (11.2 kDa; 66.9 kDa as hexamer), lanes 2 and 8: Molecular Weight Marker (Thermo Scientific PageRulerTM Plus Prestained Protein Ladder), lane 3: 2 µg ribosomal protein S1 (61.1 kDa), lane 4: 5 µg RNA polymerase (α: 36.5 kDa, β: 150.6 kDa, β‘: 155.1 kDa, ω: 10.2 kDa), lane 5: 5 µg Poly(A)polymerase (53.8 kDa), lane 6: 10 µg Rho (47.0 kDa; 282.0 kDa as hexamer), lane 7: 10 µg Translation elongation factor Tu EF-Tu (43.2 kDa). On panels A, B and C signals directly due to the hexamer of Hfq are marked with a red asterisk, signals due to Hfq interacting with another protein are marked with a blue asterisk (**A** and **C**). In the case of S1 the anti-Hfq antibody is reacting with S1 (**B**). The sizes of the molecular weight markers, (visible on membranes after transfer) are noted on all three gels. The EF-Tu-Hfq complex is indicated by an arrow. It should be noticed that several markers are revealed on the far western blot.

The interaction of Hfq with several proteins susceptible to interact with Hfq either directly (EF-Tu) or together with RNA (PAP, RNA polymerase, Rho) and also ribosomal protein S1 was tested by a far-western experiment. Purified proteins were separated on three identical SDS gels, two of which were blotted to nitrocellulose membranes. After denaturation and renaturation of the proteins, one membrane was incubated with Hfq (far western blot experiment) (Fig. 3A) and then after extensive washing, both were treated with polyclonal antibodies against Hfq (the second membrane serves as a direct western blot control) (Fig. 3B,C). The third gel was stained with Coomassie blue (Fig. 3D). Note that Hfq migrates as a hexamer (66.9 kDa) on SDS gels. The direct western blot experiment (Fig. 3B) revealed the presence of Hfq (red asterisk, lane 3) in the preparation of S1. A trace of Hfq is also detected in the preparations of RNA polymerase, PAP, EF-Tu and Rho when the blot is over exposed (Fig. 3C), most probably because Hfq is a known false positive, able to bind to Ni-NTA agarose columns in the absence of a H6 tag. Signals corresponding to the size of S1 and hexameric Hfq were detected on western (Fig. 3 panels B–C) and far western (panel A) blots in the case of S1 (indicated by blue and red stars Fig. 3A–C lane 3), possibly because the purified Hfq used for making the antibodies was contaminated with S1 protein (see Materials and Methods). Alternatively, both S1 and Hfq have RNA binding motifs, which could be recognized by the same antibodies. Under the experimental conditions employed, Hfq interacts weakly with high molecular weight forms of RNA polymerase β, β′ subunits, Rho (probably as a hexamer but not the 47 kDa monomeric form) and aggregated forms of poly(A) polymerase (but not the 53.8 kDa monomeric form). On the other hand a discrete band signaling an interaction between monomeric (44 kDa) EF-Tu and Hfq is detected on the far western blot (Fig. 3A lane 7 indicated with an arrow), which is not seen on the direct Western (panels B and C). This confirms a protein-protein interaction between EF-Tu and Hfq as detected by TAP-tag and DSP cross-linking. The signals detected with the high molecular weight “aggregate” forms of the other proteins are difficult to interprete as the exact composition is unknown. In the case of the ribosomal protein S1, no conclusion can be drawn because of the cross reactivity of the anti-Hfq polyclonal antibody with S1, although the relative intensity of the bands could suggest an interaction between these proteins. A positive signal in the far-western blot indicates whether two proteins bind to each other directly, but the intensity of the signal depends upon the level of renaturation of the proteins on the membrane rendering this method non-quantitative. Other techniques are required to further evaluate the interactions detected by far-western (Fig. 3A).

### Microscale Thermophoresis Experiments

**Figure 4:**
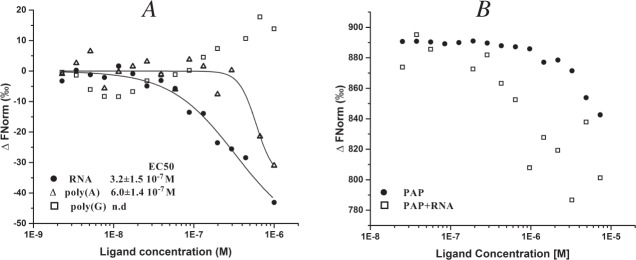
Ligand binding to HfqH6. Binding curves are derived from the specific change in the thermophoretic mobility upon ligand titration to a constant 100 nM concentration of HfqH6. (**A**) The curves show binding affinities of HfqH6 for poly(G) (open squares), poly(A) (open triangles) or RNA (filled circles). For determination of the EC50, the Hill Model was used that is included in the NT Affinity Analysis software. (**B**) The curves show the binding of PAP to HfqH6 in the presence (open squares) or in the absence of RNA (filled circles). Total RNA (heterologous in size and form), poly(A) and poly(G) were used with 1 kb as a crude approximation for calculation. Thereby, if we consider a 50–100 nt RNA long, the binding constants return the range typically reported for Hfq.

Before performing Microscale Thermophoresis (MST) experiments, we verified the thermal stability and the secondary structure content of the Hfq constructs by differential scanning calorimetry (DSC) and circular dichroism (CD) (data not shown). Microscale Thermophoresis estimates the modifications of molecular movement along temperature gradients due to changes in conformation or environment and can be used to measure protein-ligand interactions^46^. We first determined the binding affinity of HfqH6 for *E. coli* total RNA, poly**(**A**)** and poly**(**G**)** by MST (Fig. 4A). We used in these experiments total RNA in order to be the closest to the *in vivo* situation, where Hfq binding sites, are distributed throughout the RNA and the majority of RNA is rRNA. As total RNA is heterogeneous in size and sequence, and commercial poly**(**A) and poly**(**G**)** samples also range in size, an average size 1 kb of these molecular species was chosen for further calculation as previously used^47^. While no significant binding of Hfq to poly(G) was found, the half maximal effective concentration (EC50 values) for RNA and poly**(**A**)** were both less than micro-molar (3.2 ± 1.4 10^−7^ M and 6.0 ± 1.4 10^−7^ M, respectively) (Fig. 4A). However, the cooperativity of the binding was found to be quite different, as measured by the Hill coefficient (1.0 for RNA and 4.0 for poly(A)). This is probably due to the heterogeneity of binding molecules present in the total RNA, with for instance the absence of long stretches of A residues in total mRNA limiting the cooperativity. These results confirm the previous observations for the preferential cooperative binding of Hfq to poly(A)^48^.

We then tested the binding affinity of HfqH6 for PAP (Fig. 4B). A binding was observed with an affinity (EC50), which was estimated at less than 10 µM. A similar affinity of binding to PAP was observed with non-tagged Hfq (data not shown). In the presence of total RNA we observed a diminution of the apparent affinity of about 10-fold that was associated with a significant signal dispersion, due to the heterogeneous RNA used (Fig. 4B). This decrease in affinity could be explained by a transient masking of binding sites for PAP on Hfq by some RNA molecules. It suggests that RNA is unlikely to be necessary for formation of a binary PAP and Hfq complex but that its presence modifies their interaction as shown by the observation that RNase treatment of the HfqTT complex, disrupted the binding of Hfq to PAP. Similar effects of total RNA on the interaction of S1 and RNA polymerase with Hfq were observed but with affinity levels requiring too high concentrations of ligand to be quantifiable with this technology (data not shown).

It has previously been shown that the Rho factor physically interacts with Hfq conferring an anti-termination activity to the tandem complex on mRNA^14^. Our TAP-tag and DSP crosslinking experiments confirm this interaction and also show an RNA dependence. When we attempted to measure this interaction *in vitro* by MST, no interaction was detected between Hfq and Rho either in the absence or presence of total RNA, whatever partner was labelled. This result was disappointing considering that our data and previous results strongly imply that RNA is required to mediate or stabilize the Rho-Hfq interaction *in vivo*. In addition using the purified proteins, we only observed a faint interaction between Hfq and the hexameric form of Rho by far western blotting. These negative results with purified proteins could imply that other components may be required to stabilize the interaction (another protein, ATP, or a specific RNA underrepresented in the total RNA used in this MST assay and absent in far western).

## Discussion

Characterizing protein-protein interactions is challenging in the case of Hfq since this protein is very abundant and has been found associated with ribosomes, DNA and RNA^6,7,43^. Furthermore, many studies have demonstrated the association of Hfq with the various machines of RNA metabolism, for its synthesis, translation and degradation^1,2,5^. Systematic analysis of protein-protein interactions in *E. coli* had implied that Hfq interacts with a large number of partners^17,18^. Our results support and extend these observations. By using TAP-tag and *in vivo* crosslinking, we identified 96 and 205 partners (non ribosomal proteins), respectively, with 55 in common (Fig. 2). Out of these 55 partners, only 11 are also present in the INTACT database. Fifteen partners are found by one or other of our approaches and are present in the INTACT database (Table S5). Overall these results show that the technologies employed appear to dictate a large subset of the proteins identified especially when carried out in different laboratories. However the broad overlap in our set of interacting proteins by the two methods, associated with the previous data both from global analyses and studies on individual proteins supports the identification of a core set of Hfq-interacting proteins. As well as confirming the interaction between Hfq and the main players in RNA metabolism, in particular we demonstrate the importance of RNA in mediating the binding of Hfq with the numerous enzymes of RNA synthesis, namely those where essentially all Hfq binding was lost after RNase A treatment. The role of RNA in one of these Hfq complexes was investigated by MST. At least in the case of PAP, there was evidence that RNA and PAP compete for the same site on Hfq. Three RNA binding sites on the Hfq hexamer were defined previously and called the proximal, distal and the rim RNA binding surfaces^49,50^. PAP activity is stimulated by Hfq, and this stimulation is correlated with Hfq binding to the RNA^27,51,52^. Localization of the contact sites between Hfq and PAP and identification of the Hfq RNA binding sites involved in this interaction represent an interesting task for further studies.

As in previous studies, we have also detected interactions of Hfq with many ribosomal proteins and some modification factors acting on ribosomal RNA, by both TAP-tag and DSP crosslinking. However since the majority of the ribosomal candidates were also bound to the TAP-tag only control, they are possible false positives. The interaction between Hfq and the ribosome and ribosome modification factors merits further study.

Our results also reveal several strong candidates, which interact with Hfq in the absence of RNA. First amongst these is EF-Tu, an abundant protein with both tRNA and ribosome binding activities. A weak signal is detected by far-western supporting a direct binding of Hfq to EF-Tu. Any consequences of an interaction of Hfq and EF-Tu on cell metabolism merits investigation. Proteins with important roles in DNA function also interact with Hfq such as RecA and GyrA. As in the case of the ribosome, direct consequences of their interaction with Hfq need to be explored. We speculate that the Hfq-RecA interaction may be one clue to explain the high sensitivity of the *hfq* mutant to ultraviolet light^6^. Several nucleoid associated proteins were found associated with Hfq. CbpA, Dps, HUα and β (DbhA and B), IciA, StpA and H-NS were all previously shown to copurify with Hfq^53^. Other high scoring candidates as novel Hfq interaction partners, include YhbS a putative acetylase, and various metabolic enzymes like AceA (isocitrate lyase), or some involved in fatty acid metabolism, FabB and LpxD, or ATP synthase subunits. Their potential to interact with Hfq could reflect a novel role of Hfq and/or have unforeseen consequences on the ribocontrol by Hfq of gene expression in bacteria. It remains to be determined whether the novel interactions identified in this work depict functional consequences on either or both of the interacting partners. RNA binding proteins often exhibit degenerate binding sites allowing a dynamic cycling of ligands which can easily enter and leave complexes or be temporarily sequestrated. In this context the large number of high scoring candidates for a non-RNA dependent interaction with Hfq are a starting point to look for other roles of Hfq. Although it must be admitted that some of these candidates, like EF-Tu, could be false positives just because of their high cellular abundance, others like YhbS, could interact with Hfq in the presence or in the absence of Hfq depending on the growth phase.

An important result from our study is that the majority of the interactions of Hfq with the proteins of RNA metabolism, from synthesis to degradation (RNA polymerase, Rho, PAP, Pnp, RNase E, RNase R) are essentially dependent upon RNA. The next question will be to determine whether specific sRNA or RNA fragments are part of these ribonucleoparticles. It was recently shown that Crc affects the binding of the sRNA PrrF2 to the proximal side of Hfq in *Pseudomonas aeruginosa*^54^. It is possible that some of the numerous interactants in *E. coli* may target Hfq to specific sRNAs or mRNAs, conversely the activity of these interactants may also be modified with significant consequences on transcription in the case of RNA polymerase or DNA-binding histone-like proteins. In agreement with this hypothesis Hfq was shown to affect mRNA levels independently of mRNA degradation^2^. Despite its wide spectrum of known functions, the capacity of Hfq to interact with many proteins has to be taken into account to reevaluate the global role of Hfq, which may be under appreciated.

## Supplementary information

Table S1

Table S2

Figure S1

Table S4

Table S3

Table S5

## Data Availability

The datasets generated during and/or analysed during the current study are available in the supplementary information and any additional information regarding the data in the paper is available from the corresponding author upon reasonable request.
