# Supplementary material for: Identification of protein-protein and ribonucleoprotein complexes containing Hfq
Source: Sci Rep. 2019 Oct 1;9:14054. doi: 10.1038/s41598-019-50562-w (PMC6773851; doi:10.1038/s41598-019-50562-w)
Supplement: Supplementary file 1 — Table S1 [file 41598_2019_50562_MOESM1_ESM.docx]

**Table S1: Strains and plasmids used in this study.**

**Strains: Relevant genotype References**

ENSO : former name HfrG6Δ12 wt [^1^](#_ENREF_1)

IBhfq95Δ*hfq* : ENSO *hfq::lacZ* Δ*hfq*  [^2^](#_ENREF_2)

**Plasmids:**

pACYC184 : medium copy replicon, (cat and tc) [^3^](#_ENREF_3)

pCA24N : high copy number plasmid (*lacIq,* cat) [^4^](#_ENREF_4)

pCAhfq : (cat, *lacIq*, *hfq* under inducible T5-lac promoter) this work

pBS1479 :TAP-tag peptide (amp) [^5^](#_ENREF_5)

pCAhfqTT : pCA24N derivative (cat, *lacIq*, *hfq*TT from this work

inducible T5-lac promoter)

pCATT : pCA24N derivative (cat, *lacIq*, TAP-tag peptide from this work

inducible T5-lac promoter)

pTX381 : *miaA-hfq-hfIX'*; (*hfq* gene with its own p3 promoter) cloned [^6^](#_ENREF_6)

between *Bam*HI and *Hind*III sites of pACYC184

phfqTT : pTX381 derivative (cat, *hfq*TT under the control of its own p3 this work promoter)

phfqH6 : pTX381 derivative (cat, *hfq*H6 under the control of its own p3 this work promoter)

**Oligonucleotides:**

| Name | Sequence | Comments |
| --- | --- | --- |
| JC371 | CACACAGAATTCATTAAAgaggagAAATTAACT**ATGGCTAAGGGGCAATCTTTACAAGATCCG** | lower case : *Bse*RI restriction site ; **bold** : hfq sequence |
| JC372 | GCTAATTaagcttGGCTGCAGTCGAG**TTATTCGGTTTCTTCGCTGTCCTGTTGCG** | lower case : *Hind*III restriction site ; **bold** : hfq sequence |
| JC375 | *CGGCTATGAAATTCTTTTTCCATCTTCT***TTCGGTTTCTTCGCTGTCCTGTTGCGC** | *italic*: TAPTag sequence  **bold** : hfq sequence |
| JC376 | **GCGCAACAGGACAGCGAAGAAACCGAA***AGAAGATGGAAAAAGAATTTCATAGCCG* | **bold** : hfq sequence ;  *italic*: TAPTag sequence |
| JC320 | GCTCACTCATTAGGCACCCCAGGC | in pCAhfq |
| JC377 | GGTAAAGCTATTCATCCAGCAGGCCTC | in pBS1479 |
| JC390 | CACACAGAATTCATTAAAgaggagAAATTAACT**ATGGAAAAGAGAAGATG** | lower case : *Bse*RI restriction site ; **bold** : TAPTag sequence |
| Hfq390-for | CTCATCACAGTAACAACGCCGG |  |
| Taptag-rev1 | CTGTTCAGCTACTGACGGGGTG |  |
| BamHIpAC-for | CGATGCGTCCGGCGTAGAggatcc | lower case : *Bam*HI restriction site |
| Hfq411-rec | CCGGCGTTGTTACTGTGATGAG | Reverse complement of Hfq390-for |
| JC394 | GGACAGCGAAGAAACCGAACTCGAGcaccaccaccaccaccacTAAAGCTTAATTAGCTGAGCTTGG | Lower case : His6 |
| JC395 | CCAAGCTCAGCTAATTAAGCTTTAgtggtggtggtggtggtgCTCGAGTTCGGTTTCTTCGCTGTCC | Reverse complementary of JC394 |

**Methods: Construction of plasmids.**

pCAhfqTT and pCATT: are derivatives of pCA24N [^4^](#_ENREF_4) pCAhfqTT expresses HfqTT from the T5-lac promoter while pCATT expresses just the TAP-tag peptide. First, we performed a PCR on *E.coli* chromosomal DNA to amplify *hfq* ORF with oligos JC371 and JC372. After digestion with *Bse*RI and *Hind*III the fragment was cloned between the same sites of pCA24N giving the pCAhfq plasmid. Then two PCR were performed with (i) JC375 and JC320 and pCAhfq plasmid DNA to amplify the *hfq* coding region and (ii) JC376 and JC377 with the pBS1479 plasmid DNA to amplify the TAP-Tag region. We then annealed these two PCR fragments and amplified the product using oligos JC320 and JC377. The final PCR product was digested with *Xho*I and *Hin*dIII (there is a *Hin*dIII site immediately downstream the TAP-tag stop codon) and cloned into the same sites of pCAhfq to give the pCAhfqTT plasmid. In order to get a control plasmid harboring only the TAP-tag under the control of the T5-lac promoter, we performed a PCR on pBS1479 plasmid with oligos JC377 and JC390 (harbouring the *Bse*RI restriction site), digested by *Bse*RI and *Hind*III and cloned the resulting fragment into the same sites of pCAhfqTT to give the pCATT plasmid.

phfqTT: is a derivative of pTX381, harbouring *hfq* with the TAP-tag under the control of its own P3 promoter. We performed a PCR on pCAhfqTT to amplify the TAP-tag fused to *hfq* with oligos hfq390-for and taptag-rev1 and on pTX381 to amplify the upstream region and the 3' region of *hfq* with oligos BamHIpAC-for and Hfq411-rev. These two fragments exhibiting a common segment of 22bp were combined by PCR using oligos BamH1pAC-for and taptag-rev1. The final amplified DNA fragment was digested by *Bam*HI and the resulting fragment was cloned in the *Bam*HI site of pACYC184 to give phfqTT.

phfqH6: is a derivative of pTX381, harbouring *hfq* under the control of its own promoter P3. Two PCR were performed on (i) plasmid pTX381 with oligos JC395 and BamH1pAC-for to amplify the upstream region of the N-terminal region of *hfq* and (ii) plasmid pCAhfq with JC394 and Taptag-rev1 to amplify the C-terminal region of Hfq. A final PCR combining these two fragments with oligos BamH1pAC-for and Taptag-rev1 was performed, digested with *Bam*HI and the resulting DNA fragment was cloned in the *Bam*HI site of pACYC184 to give phfqH6.

1 Dreyfus, M. What constitutes the signal for the initiation of protein synthesis on *Escherichia coli* mRNAs? *J Mol Biol* **204**, 79-94 (1988).

2 Ziolkowska, K. *et al.* Hfq variant with altered RNA binding functions. *Nucleic Acids Res* **34**, 709-720 (2006).

3 Chang, A. C. Y. & Cohen, S. N. Construction and characterization of amplifiable multicopy DNA cloning vehicles derived from the P15A cryptic. *J. Bacteriol.* **134**, 1141-1156 (1978).

4 Kitagawa, M. *et al.* Complete set of ORF clones of *Escherichia coli* ASKA library (A Complete Set of *E. coli* K-12 ORF Archive): Unique Resources for Biological Research. *DNA Res.* **12**, 291-299 (2005).

5 Puig, O. *et al.* The tandem affinity purification (TAP) method: a general procedure of protein complex purification. *Methods* **24**, 218-229 (2001).6 Tsui, H.-C., T., Leung, H.-C., E. & Winkler, M. E. Characterization of broadly pleiotropic phenotypes caused by an *hfq* insertion mutation in *Escherichia coli* K-12. *Mol. Microbiol.* **13**, 35-49 (1994).
