# Supplementary material for: Identification of protein-protein and ribonucleoprotein complexes containing Hfq
Source: Sci Rep. 2019 Oct 1;9:14054. doi: 10.1038/s41598-019-50562-w (PMC6773851; doi:10.1038/s41598-019-50562-w)
Supplement: Supplementary file 2 — Table S2 [file 41598_2019_50562_MOESM2_ESM.docx]

**Table S2: The HfqTT and HfqH6 proteins are active in the control of *rpoS* and *ptsG* expression.**

**A)** **Control of *rpoS* expression.**

Strain Plasmid β-galactosidase activity

RO91 Δ*hfq* pACYC184 15.6 ± 0.4

RO91 Δ*hfq* pTX381 (hfq) 143.3 ± 1.4

RO91 Δ*hfq* phfqTT (hfqTT) 183 ± 10

RO91 Δ*hfq* phfqH6 (hfqH6) 120.2 ± 3

RO91 *hfq*^+^ pACYC184 83.8 ± 3.2

RO91 and RO91 ∆*hfq* strains containing a chromosomal *rpoS742::lacZ* fusion [^1^](#_ENREF_1) transformed with pACYC184 (empty vector), or pACYC184 derivatives expressing either Hfq (pTX381), HfqH6 (phfqH6) or HfqTAP-tag (phfqTT) were grown in exponential phase (OD_650_=0.5). Cells were disrupted by sonication and β-galactosidase activity was expressed as nmol ONPG hydrolysed/min/mg of total soluble cell proteins [^2^](#_ENREF_2).

**B) Control of *ptsG* expression.**

α-MG - + + + + +

IPTG (M) 0 0 10^-5^ 5 10^-5^ 10^-4^ 10^-3^

ENSO pACYC184 chrom ++ ++ ++ ++ ++ ++

IBhfq95 Δ*hfq* pACYC184 - ++ ± ± ± ± ±

IBhfq95 Δ*hfq* pCAhfq wt ++ ++ ++ ++ + -

IBhfq95 Δ*hfq* pCATT - ++ ± ± ± ± -

IBhfq95 Δ*hfq* pCAhfqTT hfqTT ++ + ++ ++ + -

IBhfq95 Δ*hfq* pTX381 wt ++ ++ ++ ++ ++ ++

IBhfq95 Δ*hfq* phfqTT hfqTT ++ ++ ++ ++ ++ ++

IBhfq95 Δ*hfq* phfqH6 hfqH6 ++ ++ ++ ++ ++ ++

The cellular response to glucose-specific phosphoenolpyruvate phosphotransferase system (PTS)-dependent phosphosugar stress is mediated by SgrS sRNA [^3^](#_ENREF_3). Intracellular accumulation of glucose 6-phosphate or treatment of cells with the non-metabolizable glucose PTS substrate α-methyl glucoside (α-MG) led to rapid degradation of *ptsG* mRNA by a mechanism requiring RNase E and Hfq [^4^](#_ENREF_4). Strain ENSO (wt) and IBhfq95Δ*hfq* (*hfq*) were transformed with the empty vectors (pACYC184 and pCATT), or their derivatives expressing either wt Hfq (pTX381 and pCAHfq), His-tagged Hfq (phfqH6) or HfqTAP-tagged (phfqTT). Growth inhibition by 0.2% α-MG was tested on LB plates containing chloramphenicol 25μg/ml in the presence of increasing amount of IPTG to induce the expression of the Hfq variants cloned in the pCA24N derivatives. Hfq is expressed from its own promoter in pTX381, phfqTT and phfqH6. The growth is estimated : ++ strong growth, + medium growth, ± weak growth, – no growth. Lack of growth of pCAhfq, pCATT and pCAhfqTT containing cells with 10^-3^M IPTG suggests that the high level of TAP-tag is toxic for the cell.

1 Caillet, J., Gracia, C., Fontaine, F. & Hajnsdorf, E. *Clostridium difficile* Hfq can replace *Escherichia coli* Hfq for most of its function. *RNA* **20**, 1567-1578, doi:10.1261/rna.043372.113 (2014).

2 Ziolkowska, K. *et al.* Hfq variant with altered RNA binding functions. *Nucleic Acids Res* **34**, 709-720 (2006).

3 Rice, J. B. & Vanderpool, C. K. The small RNA SgrS controls sugar-phosphate accumulation by regulating multiple PTS genes. *Nucleic Acids Res* **39**, 3806-3819, doi:10.1093/nar/gkq1219 (2011).

4 Morita, T., Kawamoto, H., Mizota, T., Inada, T. & Aiba, H. Enolase in the RNA degradosome plays a crucial role in the rapid decay of glucose transporter mRNA in the response to phosphosugar stress in *Escherichia coli*. *Mol Microbiol* **54**, 1063-1075 (2004).
