# Supplementary material for: Identification of protein-protein and ribonucleoprotein complexes containing Hfq
Source: Sci Rep. 2019 Oct 1;9:14054. doi: 10.1038/s41598-019-50562-w (PMC6773851; doi:10.1038/s41598-019-50562-w)

Identification of protein-protein and ribonucleoprotein complexes containing Hfq

Joël Caillet[1], Bruno Baron[2], Irina V. Boni[3], Célia Caillet-Saguy[4] and Eliane Hajnsdorf[1*]

[1] UMR 8261, CNRS, Université de Paris, Institut de Biologie Physico-Chimique, 75005 Paris

[2] Institut Pasteur, Plateforme de Biophysique Macromoléculaire, Center for Technical Resources and Research (C2RT), 25-28 rue du Dr Roux, 75724 Paris cedex 15, France.

[3] Shemyakin-Ovchinnikov Institute of Bioorganic Chemistry RAS, 117997, Moscow, Russia

[4] Institut Pasteur, Laboratoire Récepteurs-Canaux, Département de Neuroscience, 25-28 rue du Dr Roux, 75724 Paris cedex 15, France.

*Corresponding author

Figure S1; is the uncropped version of Figure 1

Fig. 1: In vivo DSP crosslinking and analysis of Hfq-containing complexes.

After addition of DSP to IBhfq95 cells containing the pHfqH6 plasmid (lanes 5-9), or the empty vector (lanes 1-4) followed by lysis and enrichment of HfqH6 containing complexes on Ni-NTA agarose columns, the column fractions were submitted to DTT treatment (lanes 2, 4, 6 and 8), or not (lanes 1, 3, 5 and 7), and loaded on SDS PAGE. Lanes 1-4 are negative controls, and lanes 5-8 correspond to cells containing the pHfqH6 plasmid. Figure 1 shows only lanes 5 and 6. Strips were cut and analysed by MS for lanes 1, 2 and 5, 6

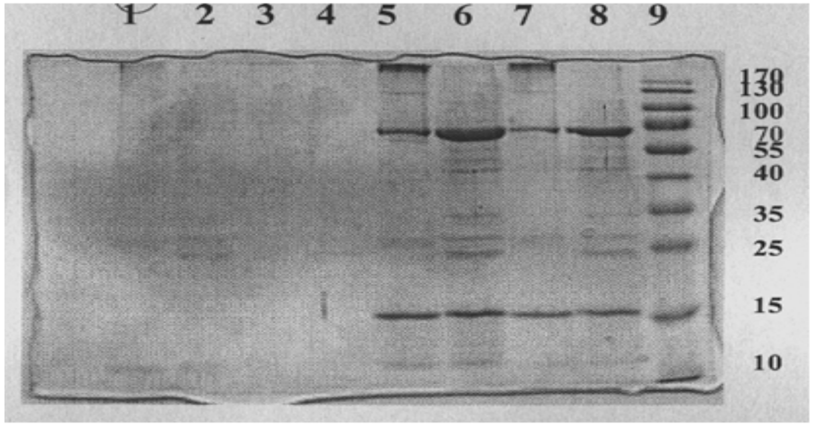

1= Δhfq/pACYC184, fraction2, +DSP, -DTT
2= Δhfq/pACYC184, fraction2, +DSP, +DTT
3= Δhfq/pACYC184, fraction3, +DSP, -DTT
4= Δhfq/pACYC184, fraction3, +DSP, +DTT
5= Δhfq/phfqH6, fraction2, +DSP, -DTT
6= Δhfq/phfqH6, fraction2, +DSP, +DTT
7= Δhfq/phfqH6, fraction3, +DSP, -DTT
8= Δhfq/phfqH6, fraction3, +DSP, +DTT

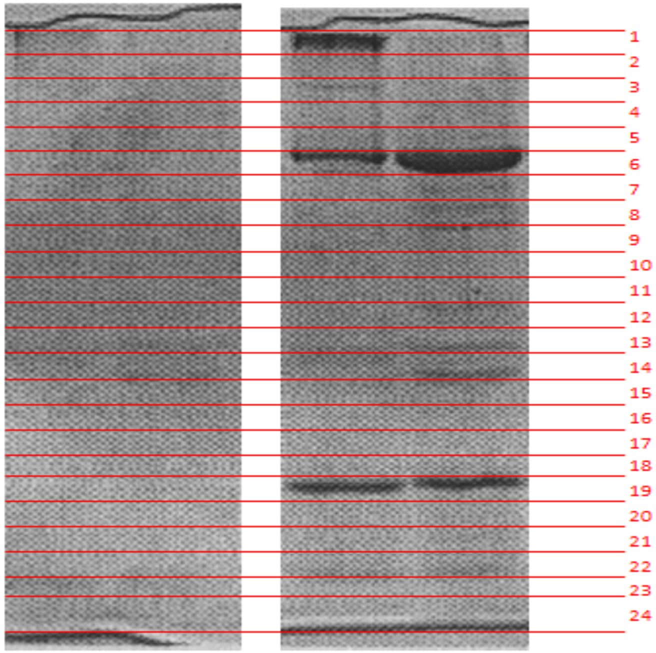

Supplement: Supplementary file 3 — Figure S1 [file 41598_2019_50562_MOESM3_ESM.pdf]
